# Disrupted Brain Functional Network Topology in Essential Tremor Patients With Poor Sleep Quality

**DOI:** 10.3389/fnins.2022.814745

**Published:** 2022-03-10

**Authors:** Jiaxin Peng, Jing Yang, Junying Li, Du Lei, Nannan Li, Xueling Suo, Liren Duan, Chaolan Chen, Yan Zeng, Jing Xi, Yi Jiang, Qiyong Gong, Rong Peng

**Affiliations:** ^1^Department of Neurology, West China Hospital, Sichuan University, Chengdu, China; ^2^Department of Radiology, Huaxi MR Research Center (HMRRC), West China Hospital, Sichuan University, Chengdu, China

**Keywords:** essential tremor, poor quality of sleep, functional connectome, graph theory, brain network

## Abstract

Sleep disturbances, especially poor quality of sleep (QoS), are common among essential tremor (ET) patients and may have adverse effects on their quality of life, but the etiology driving the poor QoS in these individuals remains inadequately understood. Few data are available on the neuroimaging alterations of ET with poor QoS. Thirty-eight ET patients with poor QoS (SleET), 48 ET patients with normal QoS (NorET), and 80 healthy controls (HCs) participated in this study. All subjects underwent a 3.0-T magnetic resonance imaging (MRI) scan for resting-state functional MRI data collection. Then, the whole-brain functional connectome was constructed by thresholding the partial correlation matrices of 116 brain regions. Graph theory and network-based statistical analyses were performed. We used a non-parametric permutation test for group comparisons of topological metrics. Partial correlation analyses between the topographical features and clinical characteristics were conducted. The SleET and NorET groups exhibited decreased clustering coefficients, global efficiency, and local efficiency and increased the characteristic path length. Both of these groups also showed reduced nodal degree and nodal efficiency in the left superior dorsolateral frontal gyrus, superior frontal medial gyrus (SFGmed), posterior cingulate gyrus (PCG), lingual gyrus, superior occipital gyrus, right middle occipital gyrus, and right fusiform gyrus. The SleET group additionally presented reduced nodal degrees and nodal efficiency in the right SFGmed relative to the NorET and HC groups, and nodal efficiency in the right SFGmed was negatively correlated with the Pittsburgh Sleep Quality Index score. The observed impaired topographical organizations of functional brain networks within the central executive network (CEN), default mode network (DMN), and visual network serve to further our knowledge of the complex interactions between tremor and sleep, adding to our understanding of the underlying neural mechanisms of ET with poor QoS.

## Introduction

Essential tremor (ET) is a common movement disorder characterized by postural or kinetic tremor of the bilateral upper limbs, with a worldwide prevalence of approximately 1% (Haubenberger et al., 2018). Apart from showing the cardinal symptom of tremor, ET patients may experience non-negligible non-motor symptoms, such as cognitive impairment, depression, anxiety, autonomic dysfunction, and sleep disturbances (Louis, 2016; Wu et al., 2016). Over the years, many studies have demonstrated that sleep disturbances, including poor quality of sleep (QoS), insomnia, rapid eye movement (REM) sleep behavior disorder (RBD), restless legs syndrome, and excessive daytime somnolence, may occur frequently in ET patients (Ondo and Lai, 2006; Gerbin et al., 2012; Sengul et al., 2014; Rohl et al., 2016; Barbosa et al., 2017). In a recent meta-analysis of sleep disturbances in ET (Jimenez-Jimenez et al., 2020), the results from nine studies using the Pittsburg Sleep Quality Index (PSQI) to assess QoS revealed higher PSQI scores and a greater frequency of poor QoS (based on the percentage of ET patients with a PSQI score ≥ 5 points) in ET patients compared to healthy controls. Benito-Leon et al. (2013) suggested that QoS is associated with the risk of ET. Besides, poor QoS may also negatively affect patients’ quality of life (QoL). Evidence from studies of Parkinson’s disease suggests that RBD may be positively correlated with α-synucleinopathy (Jiang et al., 2017; Shen et al., 2020). Elsewhere, Aribisala et al. (2020) reported that increased daytime sleep and decreased nighttime sleep efficiency are associated with neurodegenerative diseases or small-vessel diseases in older adults. In contrast, little evidence is available concerning the possible pathophysiology changes of sleep disturbances in ET, especially in ET patients with poor QoS.

Neuroimaging, including with structural and functional modalities, has been performed on ET patients to explore the pathophysiology mechanisms at play in this patient population. Among these, resting-state functional magnetic resonance imaging (rs-fMRI) provides information on neural network activities independent of a task, reflecting the baseline fluctuations in blood oxygen level-dependent signals (Fox and Raichle, 2007). To date, rs-fMRI has been widely used to evaluate neurological and psychological diseases, such as Parkinson’s disease, epilepsy, and schizophrenia. Findings from studies exploring connectivity alterations using rs-fMRI in ET suggest that disruptions in the cerebello-thalamo-cortical network may relate to motor symptoms (Lenka et al., 2017; Nicoletti et al., 2020). So far, however, there is a relative paucity of investigations on neuro-networks associated with non-motor symptoms in ET. Additionally, few studies have explored the neural basis of cognitive impairment and depression symptoms in ET (Cameron et al., 2018; Li et al., 2021b), and, to the best of our knowledge, there is no relevant neuroimaging research related to sleep disturbances in ET. Results from rs-fMRI studies of a normative aging cohort, patients with Alzheimer’s disease, and patients with Parkinson’s disease showed that disrupted neural networks of individuals with poor QoS share similar brain regions relative to those with neurodegenerative disease, overlapping specifically in the frontal, temporal, and occipital lobe regions (Chung et al., 2017; Amorim et al., 2018). Accordingly, questions have been raised about the neural network associations between ET and poor QoS.

In the past decade, the most commonly used techniques for rs-fMRI analysis have been independent component analysis, seed-based functional connectivity, and regional homogeneity; nevertheless, none of these options can completely characterize the complex brain functional network in ET. Under the circumstances, several recent studies have applied the graph theory approach in ET to investigate the neural basis, and they showed that the brain networks of ET patients exhibit topological properties known as “small-worldness,” with high reliability (Benito-Leon et al., 2019; Yang et al., 2021). Graph theory technology defines a brain network using nodes and edges representing the parcellated brain regions of interest and the correlations within nodes, respectively, then uses integration and segregation as measurements to characterize the complex network (Suo et al., 2018). Using rs-fMRI-based functional connectome methods to explore whole-brain functional networks in ET patients with and without poor QoS may provide insights into the neural basis of prevalent non-motor symptoms.

To this end, we used a graph theory approach to identify the functional connectome alterations in the topological properties of rs-fMRI to evaluate the effect of subjective poor QoS in ET patients and explore the neural basis behind it. We hypothesized that nodes involving the central executive network (CEN) and default mode network (DMN) would manifest altered functional connectivity in ET patients with poor QoS (SleET) compared to normal ET patients (NorET) and healthy controls (HCs).

## Materials and Methods

The study was approved by the local Ethics Committee of West China Hospital in China. All participants provided written informed consent before their enrollment.

### Participants

ET patients were consecutively recruited from the outpatient clinic of the Neurology Department of West China Hospital, Sichuan University, from July 2015 to June 2021. The diagnosis of ET was made based on the 1998 consensus criteria of the clinical diagnosis of ET set by the Movement Disorder Society (Chouinard et al., 1997). Demographic information was collected, including sex, age, and years of education. Furthermore, the clinical details of tremor and the clinical ratings were gathered through face-to-face interviews. We used the Fahn-Tolosa-Marin Tremor Rating Scale (TRS) (Fahn et al., 1988), which contains the TRS-A, TRS-B, and TRS-C domains, to assess the severity of tremor. The PSQI (Buysse et al., 1989) was used for the evaluation of QoS. The Mini-Mental State Examination (MMSE) (Wang et al., 1999) was used for cognitive evaluation. Finally, the Hamilton Anxiety Rating Scale (HAM-A) (Hamilton, 1959) and the Hamilton Depression Rating Scale (HAM-D-24) (Hamilton, 1967) were used to assess mood symptoms. The study inclusion criteria for the ET patients were as follows: (a) postural and/or kinetic tremor involving the bilateral upper extremities; (b) age 20–80 years; (c) disease duration ≥ 3 years; and (d) right-handedness. Separately, the study exclusion criteria for all participants were as follows: (a) identifiable brain lesions on T1- or T2-weighted MRI; (b) presence of dystonia or bradykinesia referring to parkinsonism; (c) presence of other neurological signs or abnormal blood test results indicating secondary tremor disorders; (d) obvious stepwise tremor progression; (e) history of psychological disorders or dementia; (f) obvious depressive/anxiety complaints (with HAM-A score > 14 points or HAM-D score > 17 points) (Li et al., 2018); (g) a history of receiving anti-dementia, anti-depression, anti-cholinergic, sedation, or anti-tremor medication (including β-blockers and γ-aminobutyric acid derivatives); and (h) a history of traumatic or stressful life events within the last year. A total of 38 ET patients with PSQI scores ≥ 6 points defined as having poor QoS (SleET group) and 48 ET patients without poor QoS (PSQI scores < 6 points; NorET group) were enrolled. Results of clinical evaluations, basic blood tests, and an MRI examination were obtained during the first visit, before treatment of tremor and sleep disturbances using medication, to exclude all medication confounders.

Furthermore, 80 HCs, who were right-handed and age- and sex-matched to the enrolled ET patients, were recruited through poster advertisements or as family members or friends of the enrolled patients. We excluded individuals with a history of neurological or psychiatric disorders, especially sleep disturbances, from the HC group.

### Magnetic Resonance Imaging Data Acquisition

The MRI data of all subjects were collected using a 3-T system (Tim Trio; Siemens, Erlangen, Germany) and an 8-channel phased-array head coil. During data acquisition, each participant was asked to lay supine with their head immobilized by foam pads. Participants were given earplugs to minimize the effects of MRI scanner noise and were instructed to remain relaxed, with their eyes closed without falling asleep, and to let their thoughts come and go. Axial T1-weighted, T2-weighted, and fluid-attenuated inversion recovery images were acquired for the structural assessment to exclude subjects with space-occupying brain lesions and cerebrovascular diseases. rs-fMRI was performed with the following scan parameters: repetition time, 2,000 ms; echo time, 30 ms; flip angle, 90°; axial sections, 30 slices; slice thickness, 5 mm (no gap); field of view, 240 × 240 mm; voxel size, 3.75 × 3.75 × 5.0 mm^3^; and matrix, 64 × 64. The functional run of each scan contained 240 image volumes, and the total imaging time was 480 s. After the scanning session, two neuroradiologists verified the image quality and assessed the images for clinical abnormalities in a double-blinded manner.

### Processing of Image Data

Data processing was performed in the MATLAB 2013b software environment (MathWorks, Natick, MA, United States) using the GRETNA graph-based network analysis toolkit (National Key Laboratory of Cognitive Neuroscience and Learning, Beijing Normal University, Beijing, China)^1^ (Wang et al., 2015). The data pre-processing steps included the following: (a) conversion of DICOM data into NIFTI data; (b) removal of the first 10 time points to exclude magnetic field uniformity; (c) slice timing correction of temporal offsets; (d) head motion correction of each volume; (e) spatial normalization to the standard Montreal Neurological Institute coordinate space of 152 templates (resampled to a voxel size of 3 × 3 × 3 mm^3^); (f) spatial smoothing (6-mm full width at half-maximum Gaussian kernel); (g) detrending to remove linear trends caused by scanner drift; (h) filtering (0.01 < *f* < 0.08 Hz); (i) nuisance signal regression (whole brain, white matter, and cerebrospinal fluid) signals and motion parameters (1.0 translational and 1.0 rotational parameters); and (j) scrubbing to remove the slices and ensure the head micro-movements at the group level with a mean framewise displacement ≤ 0.5 mm (Li et al., 2021b).

### Network Construction and Analysis

The brain network (including nodes and edges) was constructed and calculated using GRETNA (Wang et al., 2015). The whole brain was divided into 116 cortical and subcortical regions of interest using automated anatomic labeling landmarks, representing a network node defined by averaged blood oxygen level-dependent signals. The edges of the network were calculated by Pearson’s correlations of the mean time series between each pair of nodes, which resulted in a 116 × 116 symmetric functional connectivity matrix for each participant. Following the conversion of the correlation coefficients to the *Z*-values by Fisher *Z*-score transformation (Zhou et al., 2007), the weighted matrix was converted into a binary and undirected matrix through a predefined threshold. The absolute Pearson’s correlation between any two brain regions exceeding the given threshold was recorded as 1 or 0 otherwise.

Furthermore, the network analysis was based on 116 × 116 weighted matrices at each sparsity threshold. The range of the sparsity was 10% ≤ *S* ≤ 34% (at 0.01 steps) to assess the effects of thresholds. The wide range of sparsity was determined to ensure the estimability of the thresholded networks for the small-worldness characteristic with sparse properties and a minimum number of spurious edges (Watts and Strogatz, 1998). Then, we calculated the area under the receiver operating characteristic curve (AUC) over the same sparsity range mentioned above, consistent with previous studies (Suo et al., 2019; Li et al., 2020a), to characterize the brain networks free of potential bias of any single threshold. This measure has shown high sensitivity in detecting the topological alterations of brain networks (Li et al., 2020b). The small-world network (high clustering and low path length) showed high global and local efficiency, with balanced integration and segregation capabilities. The properties of the network at each sparsity level were calculated using the following parameters: (a) global metrics, including the clustering coefficient (*C*_*p*_), characteristic path length (*L*_*p*_), small-worldness (σ), normalized clustering coefficient (γ), normalized characteristic path length (λ), and network efficiency [including global efficiency (*E*_*global*_) and local efficiency (*E*_*local*_)], and (b) nodal properties, including the nodal degree, nodal betweenness, and nodal efficiency.

### Statistical Analysis

The demographics and clinical characteristics were analyzed using the Statistical Package for the Social Sciences version 24.0 software program (IBM Corporation, Armonk, NY, United States). Data showing normal distribution were analyzed using a univariate one-way analysis of variance (ANOVA), followed by the use of *post hoc t*-tests between each pair of groups. Continuous variables were compared between the SleET and NorET groups using the independent-samples *t*-test, and the Mann–Whitney *U*-test was performed to evaluate data that were not normally distributed. Categorical variables were analyzed using the chi-squared test. For the comparison of the network properties of the functional connectome among the three groups, we performed non-parametric permutation tests using MATLAB, which contained a designed model of one-way ANOVA applied to all AUC values of the global and nodal network metrics; in the meantime, age, sex, and years of education were set as covariates. Randomization was repeated 10,000 times. *Post hoc* pairwise permutation tests were conducted for measures of significant group differences. Then, the Benjamini–Hochberg procedure was applied to control the false discovery rate (FDR) for multiple comparisons at a significance level of 0.05 (Benjamini et al., 2001; Genovese et al., 2002).

Subsequently, we identified region pairs that showed between-group differences of nodal efficiency among the three groups of patients to explore connections between the SleET and NorET groups. A network-based statistics (NBS) method (University of Melbourne, Melbourne, Victoria, Australia)^2^ (Zalesky et al., 2010) was used for the analysis. We included only nodes that exhibited significant between-group differences (FDR-corrected *p* < 0.05) in both nodal degree and nodal efficiency. Firstly, one-way ANOVA was performed to define a set of significant changes between the connected regions (threshold *T* = 2.85, *p* < 0.05). Then, the *post hoc t*-test was performed to compare the SleET patient group (threshold *T* = 1.76, *p* < 0.05) and the NorET patient group (threshold *T* = 1.6, *p* < 0.05) with the HC group. All connections were then tested for significance using the non-parametric permutation method with 10,000 permutations. Finally, the results from NBS were visualized using the BrainNet viewer (MathWorks, Natick, MA, United States).

Finally, we conducted partial correlations to explore the relationships between significant functional network values and clinical characteristics, with age, sex, and years of education acting as covariates. The variables included were the age of onset, disease duration, TRS score, MMSE score, HAM-A score, HAM-D score, and PSQI score.

## Results

### Demographic and Clinical Characteristics

The demographic and clinical characteristics of our study participants are summarized in Table 1. No statistically significant differences in age, sex, and years of education existed between the three groups (*p* > 0.05). Patients in the SleET group were older at disease onset (*p* = 0.013), presented with a head tremor (*p* = 0.024) and tremor at rest (*p* = 0.028) in greater numbers, and had higher total TRS (*p* = 0.012) and TRS-B domain (*p* = 0.006) scores compared to the NorET group. Considering non-motor symptoms, SleET patients had higher HAM-A and HAM-D scores (*p* < 0.001). In addition, there was no significant difference between the SleET and NorET groups in terms of disease duration, tremor asymmetry, or MMSE score (*p* > 0.05); specifically, the framewise displacement estimates were not significantly different among the three groups or between the SleET and SleET patient groups (*p* > 0.05).

**TABLE 1:** Demographics and clinical characteristics of the SleET and NorET patients and HCs.

	SleET	NorET	HC	*P* (*F*) value	
				ANOVA	SleET vs. NorET
Age (years)	52.37 ± 14.475	51.17 ± 14.913	51.46 ± 10.577	0.163 (1.875)	0.421
Sex (M/F)	13/25	19/29	29/51	0.146	0.122
Years of education	9.184 ± 4.398	11.188 ± 4.311	10.344 ± 3.845	0.084 (2.514)	0.125
Handedness (right/left)	38/0	48/0	80/0	>0.999	>0.999
Age of onset	40.03 ± 15.537	31.46 ± 15.977	–	–	**0.013**
Disease duration	12.711 ± 10.436	12.390 ± 9.215	–	–	0.879
Positive family history	19 (50.0%)	23 (47.9%)	–	–	0.853
**Tremor distribution**					
Upper limbs	38 (100%)	48 (100%)	–	–	>0.999
Head	16 (42.1%)	10 (20.8%)	–	–	**0.024**
Voice/tongue/face	11 (28.9%)	10 (20.8%)	–	–	0.329
Legs	6 (15.8%)	4 (8.3%)	–	–	0.254
Trunk	1 (2.6%)	0	–	–	0.249
**Tremor type**			–	–	
Postural tremor	38 (100%)	48 (100%)	–	–	>0.999
Kinetic tremor	11 (28.9%)	14 (29.2%)			0.915
Rest tremor	15 (39.5%)	11 (22.9%)	–	–	**0.028**
Intention tremor	11 (28.9%)	10 (20.8%)	–	–	0.329
**Tremor asymmetry**			–	–	
Left = right	17 (44.7%)	23 (47.9%)			0.207
Left > right	15 (39.5%)	12 (25.0%)			0.060
Left < right	6 (15.8%)	13 (27.1%)			0.131
TRS	25.68 ± 15.859	17.78 ± 12.680	–	–	**0.012**
TRS-A	7.16 ± 5.889	5.58 ± 4.041	–	–	0.140
TRS-B	13.45 ± 7.500	9.26 ± 6.288	–	–	**0.006**
TRS-C	4.66 ± 4.806	3.00 ± 3.742	–	–	0.072
MMSE	26.21 ± 4.344	27.65 ± 3.192	27.78 ± 1.869	0.085 (3.805)	0.081
PSQI	17.45 ± 3.531	3.90 ± 2.106	3.41 ± 3.726	**<0.001** (**228.904**)	**<0.001**
HAM-A	8.29 ± 5.909	4.19 ± 4.608	3.03 ± 2.684	**<0.001** (**17.641**)	**<0.001**
HAM-D	9.39 ± 6.197	3.88 ± 4.163	3.79 ± 3.517	**<0.001** (**18.089**)	**<0.001**
FD	0.267 ± 0.118	0.291 ± 0.112	0.313 ± 0.167	0.244 (1.425)	0.330

*FD, framewise displacement; HC, healthy control; HAM-A, Hamilton Anxiety Rating Scale; HAM-D, Hamilton Depression Rating Scale; MMSE, Mini-Mental State Examination; NorET, essential tremor with normal sleep quality; PSQI, Pittsburg Sleep Quality Index; SleET, essential tremor with poor sleep quality; TRS, Fahn–Tolosa–Marin tremor rating scale. Entries in the last column shown in bold are statistically significant with p < 0.05.*

### Alterations in Global Brain Network Properties

**FIGURE 1:**
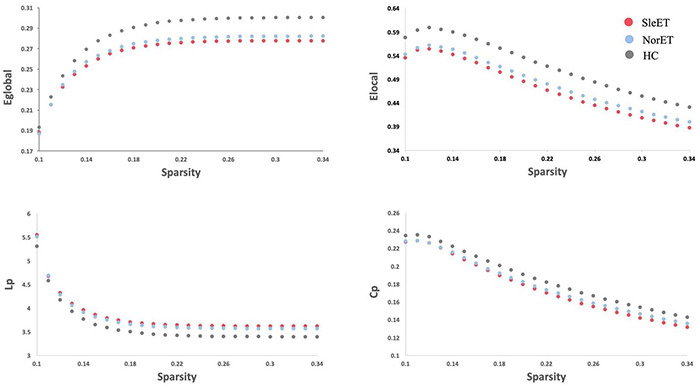
Altered global topologic properties of the brain functional connectome in SleET patients, NorET patients and healthy controls (non-parametric permutation test, *p* < 0.05). The global efficiency (Eglobal; *p* = 0.0015), local efficiency (Elocal; *p* = 0.0067), clustering coefficient (Cp; *p* = 0.0491) and characteristic path length (Lp; *p* = 0.0005) significantly differ among the three groups. *Post-hoc* tests revealed that SleET patients showed significant differences in Eglobal (*p* = 0.0003), Elocal (*p* = 0.001), Cp (*p* = 0.0131) and Lp (*p* = 0.0001). The figure shows Eglobal, Elocal, Cp and Lp of the functional networks of SleET patients, NorET patients and HC at each cost threshold (0.1–0.34, step = 0.01). SleET Essential tremor with poor sleep quality; NorET, Essential tremor with normal sleep quality; HC, Healthy controls; *p* < 0.05 with FDR correction.

All three groups of participants had a higher average clustering coefficient (γ > 1) and similar characteristic path length (λ ≈ 1), indicating small-world properties of the functional network. As for the network organization characteristics, significant differences were found in *E*_*global*_, *E*_*local*_, *C*_*p*_, and *L*_*p*_ among the three groups (FDR-corrected *p* < 0.05). Compared to the HC group, both the SleET and NorET groups showed significant reductions in *E*_*global*_ and *E*_*local*_ and a significant increase in *L*_*p*_ (*p* < 0.05), but only the SleET group showed a significant decrease in *C*_*p*_. No significant group differences were found in γ, λ, and σ. Pairwise comparisons showed no between-group global properties found in SleET vs. NorET patients (Table 2 and Figure 1).

**TABLE 2:** Global brain topological metrics showing differences between SleET and NorET patients and HCs.

Global measurements	SleET	NorET	HC	ANOVA *p* (*F*) values	*Post hoc p* (*t*) values		
					SleET vs. NorET	SleET vs. HC	NorET vs. HC
*E*_*global*_	0.0944 ± 0.0059	0.0958 ± 0.0060	0.1016 ± 0.0158	**0.0015** (**6.347**)	0.1508 (–1.048)	**0.0003** (–**2.731**)	**0.0025** (–**2.457**)
*E*_*local*_	0.1531 ± 0.0157	0.1573 ± 0.1850	0.1691 ± 0.0382	**0.0067** (**4.832**)	0.1326 (–1.135)	**0.001** (**–2.523**)	**0.0153** (**–2.024**)
*C*_*p*_	0.0552 ± 0.0071	0.0563 ± 0.0085	0.0588 ± 0.0092	**0.0491** (**3.023**)	0.2447 (–0.716)	**0.0131** (**–2.264**)	0.0563 (–1.594)
*L*_*p*_	1.3094 ± 0.0782	1.2922 ± 0.0786	1.2350 ± 0.1337	**0.0005** (**7.763**)	0.1527 (1.039)	**0.0001** (**3.236**)	**0.0023** (**2.705**)

*ANOVA, analysis of variance; C_p_, clustering coefficient; E_global_, global efficiency; E_local_, local efficiency; HC, healthy control; L_p_, characteristic path length; NorET, essential tremor with normal sleep quality; SleET, essential tremor with poor sleep quality. Entries in bold are statistically significant with p < 0.05.*

### Alterations of Nodal Brain Network Properties

Nodal metrics for the three groups of participants and *post-hoc* tests showed that the SleET and NorET groups had significant alterations in both nodal degree and nodal efficiency in the left superior frontal gyrus (SFG), dorsolateral superior frontal gyrus (SFGdor), medial superior frontal gyrus (SFGmed), posterior cingulate gyrus (PCG), lingual gyrus (LING), superior occipital gyrus (SOG), left middle occipital gyrus (MOG), and right fusiform gyrus (FFG) (FDR-corrected *p* < 0.05, ANOVA) relative to the HC group. Compared to the NorET group, the SleET group showed reductions in nodal degree (*t* = –2.441, FDR-corrected *p* = 0.0068) and nodal efficiency (*t* = –2.451, FDR-corrected *p* = 0.0074) in the right SFGmed, along with a decreased nodal efficiency in the right FFG (*t* = –2.569, FDR-corrected *p* = 0.0050) (Table 3).

**TABLE 3:** Nodal brain topological metrics showing differences between the SleET and NorET patients and HCs.

Measurements		Nodal degree				Nodal efficiency		
	ANOVA *p* (*F*) values	*Post hoc p* (*t*) values			ANOVA *p* (*F*) values	*Post hoc p* (*t*) values		
		SleET vs. NorET	SleET vs. HC	NorET vs. HC		SleET vs. NorET	SleET vs. HC	NorET vs. HC
Frontal_Sup_L	**0.0001** (**9.448**)	0.0656 (–1.538)	**0.0001** (**–3.719**)	**0.0021** (**–2.772**)	**0.0001** (**11.278**)	0.0370 (–1.933)	**0.0001** (**–3.949**)	**0.0002** (**–2.988**)
Frontal_Sup_Medial_L	**0.0017** (**6.248**)	0.044 (–1.737)	**0.0006** (**–3.260**)	**0.033** (**–1.889**)	**0.0001** (**8.904**)	0.0248 (–1.959)	**0.0001** (**–3.598**)	**0.0031** (**–2.521**)
Frontal_Sup_Medial_R	**0.0031** (**6.045**)	**0.0068** (**–2.441**)	**0.0004** (**–3.319**)	0.1058 (–1.261)	**0.0002** (**8.218**)	**0.0074** (**–2.451**)	**0.0001** (**–3.635**)	0.0173 (–2.044)
Cingulum_Post_L	**0.0037** (**5.760**)	0.2941 (–0.516)	**0.0027** (**–2.894**)	**0.0061** (**–2.514**)	**0.0001** (**10.306**)	0.2736 (–0.589)	**0.0001** (**–3.629**)	**0.0005** (**–3.365**)
Cingulum_Post_R	**0.0031** (**6.045**)	0.1373 (–1.111)	**0.0001** (**–3.728**)	**0.0021** (**–2.916**)	**0.0001** (**11.04**)	0.1085 (–1.251)	**0.0001** (**–3.943**)	**0.0009** (**–3.170**)
Lingual_L	**0.0001** (**9.061**)	0.15 (–1.040)	**0.0001** (**–3.768**)	**0.0019** (**–2.194**)	**0.0001** (**10.249**)	0.1424 (–1.076)	**0.0001** (**–3.854**)	**0.0009** (**–3.135**)
Lingual_R	**0.0001** (**10.917**)	0.0485 (–1.676)	**0.0001** (**–4.319**)	**0.0021** (**–2.833**)	**0.0001** (**11.618**)	0.0510 (–1.656)	**0.0001** (**–4.219**)	**0.001** (**–3.043**)
Occipital_Sup_L	**0.0001** (**9.285**)	0.1145 (–1.233)	**0.0001** (**–3.912**)	**0.0034** (**–2.795**)	**0.0002** (**10.326**)	0.1879 (–0.874)	**0.0001** (**–4.219**)	**0.0007** (**–3.228**)
Occipital_Sup_R	**0.0001** (**10.134**)	0.2369 (–0.708)	**0.0001** (**–3.839**)	**0.0004** (**–3.212**)	**0.0001** (**9.703**)	0.1617 (–0.998)	**0.0001** (**–3.563**)	**0.0005** (**–3.061**)
Occipital_Mid_L	**0.0001** (**9.195**)	0.441 (0.142)	**0.0009** (**–3.247**)	**0.0002** (**–3.527**)	**0.0001** (**9.169**)	0.4864 (0.0726)	**0.0006** (**–3.121**)	**0.0001** (**–3.437**)
Fusiform_R	**0.0001** (**9.528**)	0.011 (–2.299)	**0.0001** (**–4.323**)	**0.0192** (**–2.061**)	**0.0001** (**10.758**)	**0.0050** (**–2.569**)	**0.0001** (**–4.169**)	**0.0063** (**–2.444**)
Cerebellum_6_R	0.4199 (0.871)	–	–	–	**0.0186** (**4.080**)	0.0345 (–1.819)	0.0168 (–2.552)	**0.0023** (**–1.360**)

*ANOVA, analysis of variance; HC, healthy control; L, left; Mid, middle; NorET, essential tremor with normal sleep quality; Post, posterior; SleET, essential tremor with poor sleep quality; Sup, superior; R, right. Entries in bold are statistically significant with p < 0.05.*

### Alterations in Network Connectivity

**FIGURE 2:**
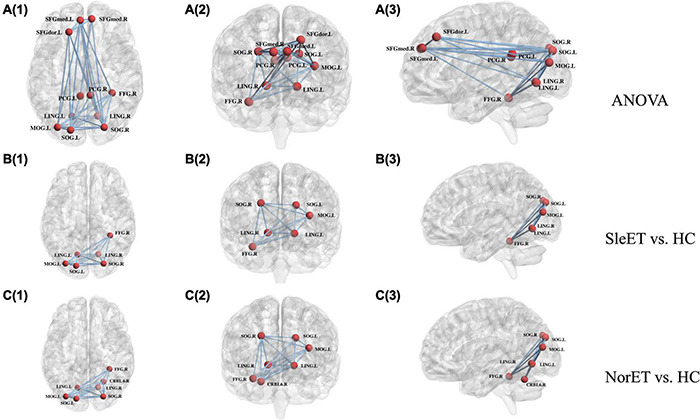
Schematic of functional connectome with significant differences, SleET, NorET with HC; between SleET and HCs, and between NorET and HC, respectively. Note that the **A(1–3)** connected network with F-statistic (one-way ANOVA, *T* = 2.85, *p* = 0.001) contains 11 nodes and 32 connections; the **B(1–3)** connected network with a T-statistic (*T* = 1.76, *p* = 0.014) contains 6 nodes and 15 connections; and the **C(1–3)** connected network with a T-statistic (*T* = 1.60, *p* = 0.008) contains 7 nodes and 15 connections. All the connections were decreased in patient subgroups compared with HCs. SleET Essential tremor with poor sleep quality; NorET, Essential tremor with normal sleep quality; HC, Healthy controls; SFGdor.L left superior frontal gyrus, dorsolateral; SFGmed.L(R) left/right superior frontal medial gyrus; PCG.L(R) left/right posterior cingulate gyrus; LING.L(R) left/right lingual gyrus; SOG.L(R) left/right superior occipital gyrus; MOG.L left middle occipital gyrus; FFD.R right fusiform gyrus; CRBL6.R right superior cerebellum.

Network alterations of the brain regions identified by the NBS tool revealed significant between-group network differences of 11 nodes and 32 connections. These nodes were mainly located in the frontal and occipital lobes and subcortical regions involving the CEN, DMN, and visual network (VIN) (Figure 2). *Post-hoc* tests showed that connection deficits of VIN were common in both ET groups relative to HCs, with a more pronounced decreased pattern present in the SleET group, while different connections in the right superior cerebellum (CRBM.6) were observed only in the NorET group relative to HCs.

### Correlation Between Topological Metrics and Clinical Characteristics

**FIGURE 3:**
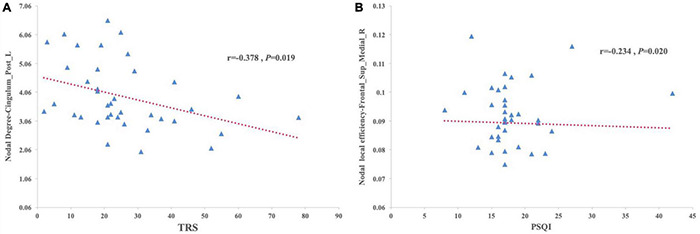
Scatterplots of correlation between the brain regions that show decreased nodal degree or decreased nodal efficiency trend with clinical characteristics in patients with SleET. **(A)** The TRS scores of SleET was negatively correlated with the nodal degree of Left posterior cingulum gyrus. **(B)** The PSQI scores of SleET was negatively correlated with the nodal efficiency of right medial superior frontal gyrus.

Partial correlation analysis was performed between the nodal degree and nodal efficiency with significant group effects and clinical variables, including the age of onset, disease duration, and the TRS and PSQI scores. The results showed that the nodal degree of the left posterior cingulum gyrus was negatively correlated with the TRS score in the SleET group (*r* = –0.378, *p* = 0.019) and the nodal efficiency of the right medial superior frontal gyrus negatively correlated with the PSQI score in the SleET group (*r* = –0.254, *p* = 0.020) (Figure 3). However, there were no significant correlations between the identified nodal properties of abnormal brain regions and clinical variables in the NorET group. Detailed information is provided in Supplementary Tables 1, 2.

## Discussion

To our knowledge, this is the first neuroimaging study to investigate the brain functional connectome in ET patients with poor QoS. Using fMRI, we found functional disruptions of single-subject brain networks in the SleET and NorET groups. Our specific findings include the following: (a) regarding the global topological organization, the SleET and NorET groups exhibited general disruption of the functional network, with a reduced ability to globally segregate, as indicated by the lower *E*_*global*_ and *E*_*local*_ and higher *L*_*p*_ values in NorET patients and by the lower *E*_*global*_, *E*_*local*_, and *C*_*p*_ and higher *L*_*p*_ values in SleET patients; (b) regarding regional topological organization, decreased nodal degree and nodal efficiency involving the CEN (left SFGdor), DMN (left SFGmed and PCG), and VIN (LING, SOG, right MOG, and right FFG) were common to both ET patient groups relative to HCs, albeit generally to a greater extent in the SleET group; (c) SleET patients showed both decreased nodal degree and nodal efficiency in the right SFGmed relative to the NorET and HC groups, and the nodal efficiency in the right SFGmed was negatively correlated with the PSQI score and the nodal degree of the left PCG negatively correlated with the TRS score; and (d) connection deficits of VIN were common to both ET subgroups relative to HCs, while different connections in the right CRBM.6 were observed only in the NorET group relative to HCs. Together, these findings further our understanding of the underlying neural mechanisms of ET and ET with poor QoS from a network perspective, and they further suggest the potential utility of a functional connectome-based approach in understanding and diagnosing ET with sleep disturbances.

A small-world network is an optimal pattern that allows a high balance of network segregation and integration. Both of our ET groups manifested decreased *E*_*local*_ values, indicating a network breakdown, resulting in a weaker local segregation ability (Suo et al., 2018). Moreover, the decreased *C*_*p*_ and *E*_*global*_ values in the ET groups suggest that a weaker small-worldness pattern leads to a less clustered brain functionality. The pathophysiology of ET has remained unclear thus far. Neuroimaging studies using the topological approach to identify the possible network malfunctions revealed that ET patients exhibit a weaker small-worldness pattern, consistent with our findings (Benito-Leon et al., 2019; Yang et al., 2021). The brain network shift toward a weaker small-worldness pattern has also been observed in patients with other neurological diseases, such as Parkinson’s disease and Alzheimer’s disease (Ruan et al., 2020), in which pathological changes such as Lewy bodies and α-synuclein or amyloid deposition are regarded as the major hallmarks. This suggests that the alteration of the topological properties of the brain in ET may have a similar neurodegenerative pathogenesis underneath. Besides the alterations of the cerebello-thalamo-cortical network, which are considered mainly responsible for the motor symptoms in ET (Mueller et al., 2017), multiple extramotor-related areas were also found with disrupted networks in ET patients; in this case, the overall brain functional network disruption could partially explain the heterogeneity of ET itself. Moreover, only the SleET patients in this study presented a decreased network clustering coefficient (decreased *C*_*p*_) compared to HCs, and this may also suggest a greater extent of disruption of the global segregation ability within this subgroup. Sleep–wake disturbances are frequent in neurodegenerative diseases with distinct global disruptions of functional connectivity, such as Alzheimer’s disease (Cordone et al., 2019) and Parkinson’s disease (Baumann, 2019). Sleep plays an essential role in the clearance of neurotoxic waste (e.g., amyloid β-peptide) (Xie et al., 2013). In a previous self-report study of elderly adults, ^18^F-fluorodeoxyglucose positron emission tomography showed that poor QoS correlated with a greater burden of cerebral amyloid β-peptide (Branger et al., 2016). In addition, sleep deprivation may lead to a reduction in the cerebral metabolic rate of glucose in a manner involving multiple motor and extramotor regions (Pak et al., 2020). Thus, the alteration of neural waste clearance or the malfunction of cerebral metabolism may underlie the global disruption of functional networks in SleET and contribute to the deterioration of QoS. However, no between-group difference was found in the global topological organization of the SleET and NorET groups under any network sparsity conditions. Several factors may have contributed to this finding. Firstly, the poor QoS in ET patients may underlie the ET pathology itself; thus, the difference in the functional network alterations between the ET subgroups can be inconspicuous at a global level. Secondly, the sample sizes of our patient groups may have limited the statistical power of this study. Future rs-fMRI investigations with larger case numbers are required.

Regarding the nodal topological alterations, though little evidence is available regarding the patterns of functional networks associated with the poor QoS of ET patients, our results showed that the nodal properties in the right SFGmed and the right FFG of the SleET group were more significant than those of the NorET group, and the nodal efficiency of the right SFGmed was negatively correlated with the PSQI score. Previous studies have identified significant changes within the DMN in ET patients (Li et al., 2021b); specifically, reduced functional networking was noted between the cerebellum and posterior DMN in ET patients (Fang et al., 2015). In our case, the nodal degree of the left PCG was found to negatively correlate with the TRS score, suggesting that disruptions within the DMN may contribute to tremor progression in ET. Furthermore, neuroimaging studies of insomnia patients revealed volume loss in the frontal cortex, hippocampus, and cingulate cortex (Spiegelhalder et al., 2015), which are partially included in the DMN. The identified right SFGmed with decreased nodal properties in SleET patients is also part of the DMN (Buckner and DiNicola, 2019). The DMN is usually activated during rest and is involved in self-referential mental thought (Raichle, 2015). Poor QoS is generally accompanied by daydreaming and rumination, which requires functioning of the DMN (Carciofo et al., 2014). In patients with insomnia, the structural covariance within the DMN was reduced and correlated with poor QoS (Suh et al., 2016), and a shorter sleep duration was also associated with a reduced functional connectivity within the DMN (Khalsa et al., 2016). Studies using seed-based analyses, dynamic functional connectivity, and graph analytic approaches demonstrated that the function of non-REM sleep depth can suggest a weakening of connectivity within the DMN (Wilson et al., 2015; Goldstein-Piekarski et al., 2020). Thus, disruption of DMN integrity may be regarded as a state marker for poor QoS (Goldstein-Piekarski et al., 2020). Our results also suggest that alterations in the nodal properties within the DMN are associated with poor QoS, as indicated by the PSQI score. Therefore, it seems that an altered integration occurring in a top-down manner in sleep regulation rather than a bottom-up manner contributes to poor QoS in ET patients. Furthermore, nodal topological alterations in the SFG may also contribute to the pathogenesis of tremor. Evidence has shown that the motor symptoms of ET might be driven by the disruption of the functional or structural connectivity in the ventral intermediate nucleus–motor cortex–cerebellum circuit (Fang et al., 2016; Benito-Leon et al., 2019; Yang et al., 2021), indicating that, aside from the cerebellum, both the motor cortex and supplemental motor area may also mediate the onset of tremor in ET.

In addition, functional connectivity alterations identified within the lingual and occipital regions in the SleET group may influence the QoS in different ways. Evidence from a prior study of normative aging people suggested that poor QoS is associated with decreased functional and structural brain connectivity with overlapping brain regions in the SFG and LING and occipital regions, in contrast to our results (Amorim et al., 2018; Neumann et al., 2020). Also, in the present study, the NBS results denoted distinct connection deficits of VIN in ET patients. How alterations of the visual circuit-related areas (i.e., the LING, SOG, right MOG, and right FFG within the VIN) interfere with QoS, either as a consequence or as a precursor in ET patients, remains unclear. Previous fMRI studies have suggested that extended tremor-related brain regions are associated with the visual cortex and parietal lobule outside the conventional tremor network of the cerebello-thalamo-motor cortical pathway (Lenka et al., 2017; DeSimone et al., 2019). Tuleasca et al. (2020) found that alterations in the network interconnectivity strength in visual association areas (e.g., the retrosplenial cortex within the occipital lobe) were associated with tremor arrest before and after the thalamotomy. Moreover, the visual-related pathway was also associated with the TRS score—that is, with tremor severity (Archer et al., 2018). It is worth noting that the SleET group exhibited greater proportions of head tremor and rest tremor and a higher level of tremor severity in our study as well. On the other hand, sleep plays an essential role in visual cortical plasticity, which has been verified in cat and rodent animal models; the identified visual cortical changes associated with sleep would shift from childhood to other stages of life (Frank, 2017). Additionally, Yu et al. (2020) observed gray matter hypertrophy within the VIN in primary insomnia patients, especially within the right FFG, and Dai et al. (2014) reported increased regional homogeneity in the FFG. This might be due to a compensatory effect of the decreased functional connectivity within the VIN. Furthermore, evidence from a sleep-related electroencephalography study showed that the EEG microstate map with a frontal-to-occipital orientation was dominant during slow-wave sleep (Xu et al., 2020), and slow-wave sleep has been associated with the maintenance of sleep (Mander et al., 2017); thus, altered functional activity of the SOG and right MOG (within the VIN) may disorganize the slow-wave sleep and affect the QoS in ET. Taken together, it is possible that poor QoS in ET patients drives VIN dysfunction and subsequently interacts with the tremor-related circuit, which may also partially explain why our SleET patients exhibited a higher level of tremor severity.

Notably, the QoS of ET patients might be affected by mood and stress as well. In our study, we excluded patients with obvious depressive or anxiety symptoms, but the HAM-A and HAM-D scores of ET patients with a normal affective state remained positively correlated with the PSQI score. Sleep quality might relate to emotional activity *via* intracortical myelination in the mid-PCG and temporal cortex, and sleep may negatively mediate affective behavior in turn (Toschi et al., 2021). In healthy adolescents, results from an rs-fMRI study showed that the fractional amplitude of low-frequency fluctuations in the left SFG is a neurofunctional marker of perceived stress (Wang et al., 2019), which is related to depressive symptoms. Besides, abnormal functional connectivity in the PCG cortices was found to be associated with both sleep and depressive problems (Cheng et al., 2018), and asymmetry of the occipital lobes was found in patients with major depressive disorder (Fullard et al., 2019), whereas our results also showed asymmetry alterations of nodal properties within the occipital lobe. Moreover, it was also reported that adequate non-REM sleep may proffer an anxiolytic benefit by restoring cingulate regions (Ben Simon et al., 2020); thus, SleET patients may suffer more anxiety than NorET patients. However, no statistically significant correlation was found between the identified brain regions and the HAM-A or HAM-D score; thus, the interaction of emotional activity and stress with QoS in ET patients may partially contribute to the alternations in the identified functional connectivity as a primary cause.

Contrary to expectations, the different connections in the right CRBM.6 observed by NBS were present only in the NorET group. Previous postmortem studies of ET patients have recorded the pathology of axonal and dendritic swellings of the cerebellum Purkinje cells, highlighting the role of the cerebellum in the pathogenesis of ET (Louis et al., 2011; Yu et al., 2012). Evidence from our previous studies has revealed functional connectivity changes within the limbic system and cerebellum in ET patients with rest tremor (Li et al., 2021a), supporting the role of the cerebellum instead of QoS in tremor symptoms. Moreover, our results from the graph theory analysis also identified a decreased nodal efficiency of the right CRBL.6 in NorET patients, while the connections and nodal properties within SleET patients showed no significant variation. This possibly reflects the compensatory improvement in the cerebellum regions of SleET patients, and the conduct of multimodal MRI analysis for further investigation should be considered.

Our study has several limitations. Firstly, this study was a cross-sectional investigation, and further longitudinal studies are needed to investigate how functional network disruptions evolve with the progression of QoS deterioration in ET. Secondly, ET patients were diagnosed based on the 1998 Movement Disorder Society criteria; thus, the ET patient group was a combination of pure ET and ET-plus patients, and the additional symptoms of the latter population (e.g., rest tremor and impaired tendon gait) may have influenced the results in some ways. Thirdly, although the PSQI is a widely used tool with high sensitivity (89.6%) and specificity (86.5%) for assessing QoS (Zhang et al., 2020), it is a subjective assessment and does not involve objective quantitative tools like polysomnography; thus, the QoS of ET patients might have been underestimated. Fourthly, the DMN and CEN also play crucial roles in cognitive processing, and our results failed to find the correlations between the DMN-related regions and the MMSE score, which may be due to the low sensitivity of the MMSE in the detection of mild cognitive impairment, so further studies with detailed cognitive evaluations are needed. Fifthly, the partial correlation of the nodal properties and clinical variables showed no significant results after covariate correction in the NorET group, which may be due to the lack of large sample sizes of ET patients and the difference in heterogeneity between the SleET and NorET groups. Finally, the topological analysis of the patients involved only rs-fMRI, so topological alterations within the brain structures of our patients remain unclear. Future investigations in ET patients with poor QoS involving multiple modules of MRI and a longitudinal design are needed.

## Conclusion

In summary, the functional connectome of both SleET and NorET patients shifted toward a weaker small-worldness on a global scale. Nodal degree and nodal efficiency were decreased mainly in the frontal and occipital lobes and some subcortical regions, involving the brain functional network of the CEN, DMN, and VIN. The nodal efficiency of the right SFGmed was positively correlated with QoS. These alterations in the topological organization of the brain functional network suggest complex interactions between tremor and sleep and have increased our understanding of the underlying neural mechanisms of ET and ET with poor QoS while also further suggesting the potential utility of a functional connectome-based approach to understanding and diagnosing ET with sleep disturbances.

## Data Availability Statement

The datasets generated for this study are available from the corresponding author by request.

## Ethics Statement

The studies involving human participants were reviewed and approved by the Ethics Committee in West China Hospital, Sichuan University. The patients/participants provided their written informed consent to participate in this study.

## Author Contributions

JP, JY, JL, XS, NL, LD, CC, and RP were performed the material preparation, data collection, and data analysis. JP wrote the first draft of the manuscript. All authors have read and approved the final manuscript, contributed to the study conception and design, and commented on previous versions of the manuscript.

## Conflict of Interest

The authors declare that the research was conducted in the absence of any commercial or financial relationships that could be construed as a potential conflict of interest.

## Publisher’s Note

All claims expressed in this article are solely those of the authors and do not necessarily represent those of their affiliated organizations, or those of the publisher, the editors and the reviewers. Any product that may be evaluated in this article, or claim that may be made by its manufacturer, is not guaranteed or endorsed by the publisher.

## Footnotes

1
https://www.nitrc.org/projects/gretna/

2
http://www.nitrc.org/projects/nbs/
