# Supplementary material for: Disrupted Brain Functional Network Topology in Essential Tremor Patients With Poor Sleep Quality
Source: Front Neurosci. 2022 Mar 10;16:814745. doi: 10.3389/fnins.2022.814745 (PMC8960629; doi:10.3389/fnins.2022.814745)
Supplement: Supplementary file 1 [file Table_1.DOCX]

**Table S1** Partial correlations of nodal efficiency and nodal degree with clinical variables in ET with poor sleep quality.

|  |  | Age of onset | Duration | TRS | HAMA | HAMD | PSQI |
| --- | --- | --- | --- | --- | --- | --- | --- |
| **Nodal efficiency** |  |  |  |  |  |  |  |
| Frontal_Sup_L | r | 0.015 | -0.012 | 0.035 | -0.043 | -0.090 | -0.024 |
|  | *p* | 0.895 | 0.915 | 0.754 | 0.697 | 0.414 | 0.825 |
| Frontal_Sup_Medial_L | r | -0.010 | 0.008 | -0.072 | 0.084 | -0.003 | -0.187 |
|  | *p* | 0.930 | 0.942 | 0.517 | 0.449 | 0.977 | 0.088 |
| Frontal_Sup_Medial_R | r | 0.036 | -0.050 | 0.043 | 0.063 | -0.018 | **-0.254** |
|  | *p* | 0.745 | 0.655 | 0.699 | 0.567 | 0.874 | **0.020** |
| Cingulum_Post_L | r | 0.049 | -0.056 | 0.089 | 0.048 | -0.028 | -0.117 |
|  | *p* | 0.661 | 0.612 | 0.423 | 0.664 | 0.798 | 0.289 |
| Cingulum_Post_R | r | 0.056 | -0.056 | 0.076 | 0.057 | -0.016 | -0.141 |
|  | *p* | 0.613 | 0.614 | 0.495 | 0.604 | 0.884 | 0.200 |
| Lingual_L | r | 0.075 | -0.076 | 0.033 | 0.059 | -0.015 | -0.168 |
|  | *p* | 0.496 | 0.494 | 0.765 | 0.594 | 0.889 | 0.128 |
| Lingual_R | r | 0.089 | -0.091 | 0.036 | 0.054 | -0.018 | -0.165 |
|  | *p* | 0.423 | 0.410 | 0.746 | 0.628 | 0.869 | 0.133 |
| Occipital_Sup_L | r | 0.087 | -0.087 | 0.014 | 0.053 | -0.008 | -0.141 |
|  | *p* | 0.432 | 0.433 | 0.901 | 0.629 | 0.944 | 0.200 |
| Occipital_Sup_R | r | 0.090 | -0.089 | 0.001 | 0.048 | -0.010 | -0.127 |
|  | *p* | 0.416 | 0.420 | 0.996 | 0.666 | 0.928 | 0.249 |
| Occipital_Mid_L | r | 0.070 | -0.073 | 0.011 | 0.047 | -0.011 | -0.126 |
|  | *p* | 0.529 | 0.511 | 0.923 | 0.674 | 0.921 | 0.254 |
| Fusiform_R | r | 0.061 | -0.065 | 0.030 | 0.062 | -0.015 | -0.144 |
|  | *p* | 0.579 | 0.554 | 0.783 | 0.578 | 0.892 | 0.191 |
| SupraMarginal_L | r | 0.073 | -0.081 | 0.031 | 0.055 | 0.006 | -0.122 |
|  | *p* | 0.511 | 0.466 | 0.777 | 0.619 | 0.959 | 0.270 |
| Cerebelum_6_R | r | 0.079 | -0.078 | 0.025 | 0.071 | 0.022 | -0.112 |
|  | *p* | 0.473 | 0.482 | 0.822 | 0.524 | 0.842 | 0.311 |
| **Nodal degree** |  |  |  |  |  |  |  |
| Frontal_Sup_L | r | 0.226 | 0.149 | -0.326 | 0.214 | 0.060 | -0.310 |
|  | *p* | 0.172 | 0.371 | 0.056 | 0.197 | 0.720 | 0.058 |
| Frontal_Sup_Medial_L | r | 0.226 | 0.055 | -0.021 | 0.166 | 0.093 | -0.061 |
|  | *p* | 0.172 | 0.745 | 0.902 | 0.320 | 0.579 | 0.717 |
| Frontal_Sup_Medial_R | r | 0.302 | 0.098 | -0.146 | 0.194 | 0.120 | -0.113 |
|  | *p* | 0.066 | 0.559 | 0.383 | 0.244 | 0.474 | 0.500 |
| Cingulum_Post_L | r | 0.240 | -0.102 | **-0.378** | 0.078 | -0.078 | -0.134 |
|  | *p* | 0.147 | 0.541 | **0.019** | 0.642 | 0.643 | 0.424 |
| Cingulum_Post_R | r | 0.218 | 0.127 | -0.527 | 0.155 | 0.182 | 0.387 |
|  | *p* | 0.188 | 0.447 | 0.061 | 0.352 | 0.275 | 0.116 |
| Lingual_L | r | 0.177 | 0.034 | 0.153 | 0.110 | 0.051 | -0.039 |
|  | *p* | 0.287 | 0.840 | 0.359 | 0.510 | 0.761 | 0.814 |
| Lingual_R | r | 0.116 | -0.045 | 0.139 | 0.149 | 0.143 | 0.144 |
|  | *p* | 0.487 | 0.790 | 0.406 | 0.372 | 0.392 | 0.389 |
| Occipital_Sup_L | r | 0.235 | -0.189 | 0.043 | -0.035 | -0.097 | -0.117 |
|  | *p* | 0.156 | 0.257 | 0.798 | 0.834 | 0.564 | 0.485 |
| Occipital_Sup_R | r | 0.335 | -0.170 | -0.067 | -0.237 | -0.346 | 0.186 |
|  | *p* | 0.141 | 0.309 | 0.690 | 0.152 | 0.133 | 0.263 |
| Occipital_Mid_L | r | 0.061 | -0.044 | -0.065 | -0.322 | -0.311 | -0.045 |
|  | *p* | 0.714 | 0.794 | 0.700 | 0.058 | 0.052 | 0.789 |
| Fusiform_R | r | 0.117 | -0.035 | 0.171 | 0.319 | 0.182 | 0.218 |
|  | *p* | 0.485 | 0.834 | 0.305 | 0.051 | 0.275 | 0.188 |
| SupraMarginal_L | r | -0.158 | -0.011 | 0.228 | 0.050 | 0.141 | -0.102 |
|  | *p* | 0.342 | 0.512 | 0.169 | 0.765 | 0.399 | 0.541 |
| Cerebelum_6_R | r | 0.059 | -0.021 | -0.096 | 0.253 | 0.312 | -0.238 |
|  | *p* | 0.062 | 0.901 | 0.567 | 0.126 | 0.057 | 0.150 |

*TRS, Fahn-Tolosa-Marin tremor rating scale; MMSE, mini-mental state examination; PSQI, Pittsburg Sleep Quality Index; HAMA Hamilton anxiety rating scale; HAMD, Hamilton depression rating scale; L left, R right, Sup superior, Post posterior, Mid middl*

*Bold numbers are statistically significant with p<0.05*
