# Supplementary material for: Disrupted Brain Functional Network Topology in Essential Tremor Patients With Poor Sleep Quality
Source: Front Neurosci. 2022 Mar 10;16:814745. doi: 10.3389/fnins.2022.814745 (PMC8960629; doi:10.3389/fnins.2022.814745)
Supplement: Supplementary file 2 [file Table_2.DOCX]

Table S2 Partial correlations of nodal efficiency and nodal degree with clinical variables in ET with normal sleep quality.

|  |  | Age of onset | Duration | TRS | HAMA | HAMD | PSQI |
| --- | --- | --- | --- | --- | --- | --- | --- |
| **Nodal efficiency** |  |  |  |  |  |  |  |
| Frontal_Sup_L | r | 0.306 | -0.214 | -0.068 | 0.132 | 0.056 | 0.138 |
|  | *p* | 0.786 | 0.144 | 0.658 | 0.386 | 0.717 | 0.365 |
| Frontal_Sup_Medial_L | r | 0.040 | 0.023 | -0.009 | 0.129 | 0.104 | 0.170 |
|  | *p* | 0.934 | 0.876 | 0.951 | 0.398 | 0.495 | 0.265 |
| Frontal_Sup_Medial_R | r | -0.012 | -0.018 | 0.174 | 0.171 | 0.183 | 0.028 |
|  | *p* | 0.518 | 0.904 | 0.254 | 0.261 | 0.230 | 0.857 |
| Cingulum_Post_L | r | 0.096 | 0.055 | 0.035 | -0.012 | -0.096 | -0.010 |
|  | *p* | 0.221 | 0.710 | 0.820 | 0.940 | 0.532 | 0.948 |
| Cingulum_Post_R | r | 0.180 | 0.040 | 0.084 | -0.008 | -0.073 | -0.077 |
|  | *p* | 0.242 | 0.788 | 0.582 | 0.958 | 0.635 | 0.614 |
| Lingual_L | r | 0.172 | 0.036 | 0.216 | 0.149 | 0.129 | -0.218 |
|  | *p* | 0.061 | 0.806 | 0.154 | 0.327 | 0.398 | 0.150 |
| Lingual_R | r | 0.296 | -0.014 | 0.287 | 0.130 | 0.066 | -0.302 |
|  | *p* | 0.072 | 0.924 | 0.056 | 0.396 | 0.666 | 0.053 |
| Occipital_Sup_L | r | 0.319 | -0.030 | 0.022 | 0.130 | 0.193 | -0.223 |
|  | *p* | 0. 695 | 0.841 | 0.887 | 0.396 | 0.204 | 0.142 |
| Occipital_Sup_R | r | 0.058 | -0.045 | -0.142 | 0.127 | 0.265 | -0.069 |
|  | *p* | 0.526 | 0. 760 | 0.354 | 0.405 | 0.079 | 0.655 |
| Occipital_Mid_L | r | 0.094 | -0.011 | -0.054 | 0.005 | 0.181 | -0.279 |
|  | *p* | 0.684 | 0.939 | 0.726 | 0.975 | 0.235 | 0.064 |
| Fusiform_R | r | 0.060 | 0.134 | 0.0301 | -0.001 | -0.071 | -0.154 |
|  | *p* | 0.134 | 0.363 | 0.838 | 0.994 | 0.643 | 0.312 |
| SupraMarginal_L | r | -0.034 | -0.102 | -0.125 | -0.096 | 0.119 | 0.093 |
|  | *p* | 0.818 | 0.489 | 0.413 | 0.530 | 0.435 | 0.545 |
| Cerebelum_6_R | r | -0.034 | -0.102 | -0.125 | -0.096 | 0.119 | 0.093 |
|  | *p* | 0.818 | 0.489 | 0.413 | 0.530 | 0.435 | 0.545 |
| **Nodal degree** |  |  |  |  |  |  |  |
| Frontal_Sup_L | r | -0.092 | -0.052 | 0.115 | 0.028 | -0.143 | 0.149 |
|  | *p* | 0.532 | 0.723 | 0.450 | 0.855 | 0.349 | 0.328 |
| Frontal_Sup_Medial_L | r | 0.050 | -0.120 | 0.238 | -0.140 | -0.182 | -0.205 |
|  | *p* | 0.738 | 0.417 | 0.116 | 0.359 | 0.231 | 0.177 |
| Frontal_Sup_Medial_R | r | 0.069 | -0.032 | 0.358 | 0.099 | 0.005 | -0.200 |
|  | *p* | 0.639 | 0.830 | 0.106 | 0.518 | 0.976 | 0.187 |
| Cingulum_Post_L | r | 0.087 | 0.024 | 0.154 | 0.114 | -0.014 | -0.048 |
|  | *p* | 0.554 | 0.874 | 0.311 | 0.454 | 0.929 | 0.756 |
| Cingulum_Post_R | r | 0.185 | 0.018 | 0.077 | 0.211 | 0.117 | 0.116 |
|  | *p* | 0.209 | 0.903 | 0.617 | 0.165 | 0.442 | 0.447 |
| Lingual_L | r | 0.270 | 0.000 | 0.102 | 0.157 | 0.077 | -0.068 |
|  | *p* | 0.063 | 0.999 | 0.505 | 0.302 | 0.616 | 0.659 |
| Lingual_R | r | 0.260 | -0.040 | 0.124 | 0.179 | 0.065 | -0.068 |
|  | *p* | 0.074 | 0.785 | 0.415 | 0.240 | 0.673 | 0.236 |
| Occipital_Sup_L | r | 0.175 | -0.019 | 0.146 | 0.134 | 0.012 | -0.180 |
|  | *p* | 0. 235 | 0.900 | 0.340 | 0.380 | 0.939 | 0.355 |
| Occipital_Sup_R | r | 0.372 | -0.100 | 0.087 | 0.163 | 0.071 | 0.141 |
|  | *p* | 0.059 | 0.500 | 0.569 | 0.283 | 0.641 | 0.715 |
| Occipital_Mid_L | r | 0.126 | 0.120 | 0.084 | 0.038 | 0.005 | -0.056 |
|  | *p* | 0.394 | 0.416 | 0.582 | 0.804 | 0.972 | 0.075 |
| Fusiform_R | r | 0.324 | 0.095 | 0.010 | 0.083 | -0.169 | -0.268 |
|  | *p* | 0.124 | 0.521 | 0.946 | 0.590 | 0.268 | 0.899 |
| SupraMarginal_L | r | -0.251 | -0.018 | 0.085 | 0.008 | 0.121 | 0.019 |
|  | *p* | 0.085 | 0.902 | 0.581 | 0.959 | 0.428 | 0.438 |
| Cerebelum_6_R | r | -0.080 | -0.157 | -0.224 | -0.047 | 0.000 | -0.119 |
|  | *p* | 0.589 | 0.287 | 0.139 | 0.760 | 0.998 | 0.869 |

*TRS, Fahn-Tolosa-Marin tremor rating scale; MMSE, mini-mental state examination; PSQI, Pittsburg Sleep Quality Index; HAMA Hamilton anxiety rating scale; HAMD, Hamilton depression rating scale; L left, R right, Sup superior, Post posterior, Mid middle*

*Bold numbers are statistically significant with p<0.05*
